# Investigating the etiology of acute febrile illness: a prospective clinic-based study in Uganda

**DOI:** 10.1186/s12879-023-08335-4

**Published:** 2023-06-16

**Authors:** Brian K. Kigozi, Grishma A. Kharod, Henry Bukenya, Sean V. Shadomy, Dana L. Haberling, Robyn A. Stoddard, Renee L. Galloway, Phionah Tushabe, Annet Nankya, Thomas Nsibambi, Edward Katongole Mbidde, Julius J. Lutwama, Jamie L. Perniciaro, William L. Nicholson, William A. Bower, Josephine Bwogi, David D. Blaney

**Affiliations:** 1 Uganda Virus Research Institute, Entebbe, Uganda; 2 College of Health Sciences, Clinical Epidemiology Unit, Makerere University, Kampala, Uganda; 3 CDC Division of High-Consequence Pathogens and Pathology, Atlanta, USA; 4 US Centers for Disease Control and Prevention, Kampala, Uganda; 5 CDC Laboratory Systems, Atlanta, USA; 6 CDC Rickettsial Zoonoses Branch, Atlanta, USA

**Keywords:** Acute Febrile illness, Malaria, Typhoid Fever, Brucellosis, Leptospirosis, Rickettsioses, Arboviral infections, Uganda

## Abstract

**Background:**

Historically, malaria has been the predominant cause of acute febrile illness (AFI) in sub-Saharan Africa. However, during the last two decades, malaria incidence has declined due to concerted public health control efforts, including the widespread use of rapid diagnostic tests leading to increased recognition of non-malarial AFI etiologies. Our understanding of non-malarial AFI is limited due to lack of laboratory diagnostic capacity. We aimed to determine the etiology of AFI in three distinct regions of Uganda.

**Methods:**

A prospective clinic-based study that enrolled participants from April 2011 to January 2013 using standard diagnostic tests. Participant recruitment was from St. Paul’s Health Centre (HC) IV, Ndejje HC IV, and Adumi HC IV in the western, central and northern regions, which differ by climate, environment, and population density. A Pearson's chi-square test was used to evaluate categorical variables, while a two-sample t-test and Krukalis-Wallis test were used for continuous variables.

**Results:**

Of the 1281 participants, 450 (35.1%), 382 (29.8%), and 449 (35.1%) were recruited from the western, central, and northern regions, respectively. The median age (range) was 18 (2–93) years; 717 (56%) of the participants were female. At least one AFI pathogen was identified in 1054 (82.3%) participants; one or more non-malarial AFI pathogens were identified in 894 (69.8%) participants. The non-malarial AFI pathogens identified were chikungunya virus, 716 (55.9%); Spotted Fever Group rickettsia (SFGR), 336 (26.2%) and Typhus Group rickettsia (TGR), 97 (7.6%); typhoid fever (TF), 74 (5.8%); West Nile virus, 7 (0.5%); dengue virus, 10 (0.8%) and leptospirosis, 2 (0.2%) cases. No cases of brucellosis were identified. Malaria was diagnosed either concurrently or alone in 404 (31.5%) and 160 (12.5%) participants, respectively. In 227 (17.7%) participants, no cause of infection was identified. There were statistically significant differences in the occurrence and distribution of TF, TGR and SFGR, with TF and TGR observed more frequently in the western region (*p* = 0.001; *p* < 0.001) while SFGR in the northern region (*p* < 0.001).

**Conclusion:**

Malaria, arboviral infections, and rickettsioses are major causes of AFI in Uganda. Development of a Multiplexed Point-of-Care test would help identify the etiology of non-malarial AFI in regions with high AFI rates.

**Supplementary Information:**

The online version contains supplementary material available at 10.1186/s12879-023-08335-4.

## Introduction

Fever without a localized cause is a common presenting symptom reported by persons seeking medical care in countries with resource-limited settings [1]. Acute febrile illness (AFI) presents as a recent onset of fever (commonly defined as a body temperature of ≥ 38.0 °C) in the absence of an obvious focus of infection and may be associated with non-specific symptoms such as headache, body rash, and muscle and joint pains [2]. Malaria, a frequent cause of AFI, contributes significantly to morbidity and mortality in sub-Saharan Africa (SSA) [3]. The non-specific presentation of various AFI etiologies makes them difficult to distinguish based on the clinical history and physical examination alone. In addition, diagnostic assays for many febrile diseases are often complex, costly, not readily available in resource-limited areas, and may have low sensitivity and specificity [4]. Furthermore, there is often limited epidemiological information on locally or regionally prevailing endemic and emerging diseases, which can complicate AFI diagnoses [5, 6]. Consequently, timely life-saving treatment and effective public health interventions may not be implemented as clinicians’ decisions are often guided by syndrome-based guidelines using empirical treatment rather than knowledge of the predominant local and regional etiologic pathogens [7].

In tropical areas of SSA, the predominant cause of undifferentiated fever has historically been assumed to be malaria [7]. During the last two decades, there has been progress towards malaria control, including the more widespread use of rapid diagnostic tests (RDTs) to exclude malaria [8]. This has led to a global decrease in malaria incidence, creating challenges for clinicians facing a growing proportion of non-malarial febrile patients and limited tests to guide diagnosis and management [6]. Consequently, there is empiric over-treatment for other potential causes of AFI with both antimalarial and antimicrobial drugs, promoting antimicrobial resistance [9].

Malaria remains a primary public health concern in Uganda. Over 95% of the country is malaria endemic, with four species identified on blood smears in children: *Plasmodium falciparum* (99% of smears)*, P. malariae* (up to 3%, often as a mixed infection with *P. falciparum*)*, P. vivax* (< 2%), and *P. ovale* (< 2%) [10]. In contrast, there is limited information on the etiology, incidence, and risk factors of non-malarial AFI-causing diseases in Uganda. Prior to this study, typhoid fever (TF) [11], arboviral diseases [12, 13], leptospirosis [14], brucellosis [15], and Spotted Fever Group (SFGR) and Typhus group rickettsioses (TGR) [16, 17] had been identified as potential non-malarial AFI diseases of major importance by the Uganda Virus Research Institute (UVRI) and the Uganda Ministry of Health (UMoH). These were hypothesized to present as undifferentiated fever leading to significant morbidity or mortality. HIV and influenza are also major contributors to the AFI disease burden. However, these have relatively well-organized, supported, coordinated public health programs and established laboratory surveillance systems, and their burden appears in the global disease estimates [18, 19].

Globally, vector-borne and nonvector-borne diseases contribute significantly to AFI [20]. The interaction of climate, environment, and population density with vectors and AFI-causing pathogens is rather complex and may impact the occurrence and distribution of the etiology of AFI. Changes in climate may affect the burden and distribution of vector-borne diseases and malaria; for example, increasing temperature provides an environment suitable for dengue and other arboviral transmissions [21].

There is a paucity of information on how climate, environment, and population density affect the occurrence and distribution of AFI pathogens in Uganda. To estimate the burden of non-malarial AFI-causing diseases, we conducted a clinic-based prospective study in three regions of Uganda differing by climate, environment, and population density.

## Materials and methods

### Study design and setting

This prospective clinic-based study enrolled participants from 28^th^ April 2011 to 19^th^ January 2013. UVRI, UMoH, and the U.S. Centers for Disease Control and Prevention (CDC) selected central, northern, and western regions for the study. These regions display intra- and inter-regional variation in environment and climate and include rural and urban areas. One district within each region was purposively selected, and a Health Centre IV (HC IV) selected within each district served as the study site. Clinic selection was based on the following criteria: 1) completeness and accuracy of clinic records; 2) representativeness of the primary care patient base in the local catchment area; 3) clinic’s resource capacity (i.e., staff size & availability, space, equipment); and 4) clinic accessibility (e.g., proximity to roadways). The environmental and geographic variation between clinics allowed a broader assessment of disease presence in Uganda. Before participant enrollment, clinic staff were trained on patient recruitment and screening, data collection, and laboratory diagnostics.

#### St. Paul’s Health Centre IV (Coordinates: 00°10.100’N 30°04.419’E; 3198 feet above sea level)

**Fig. 1:**
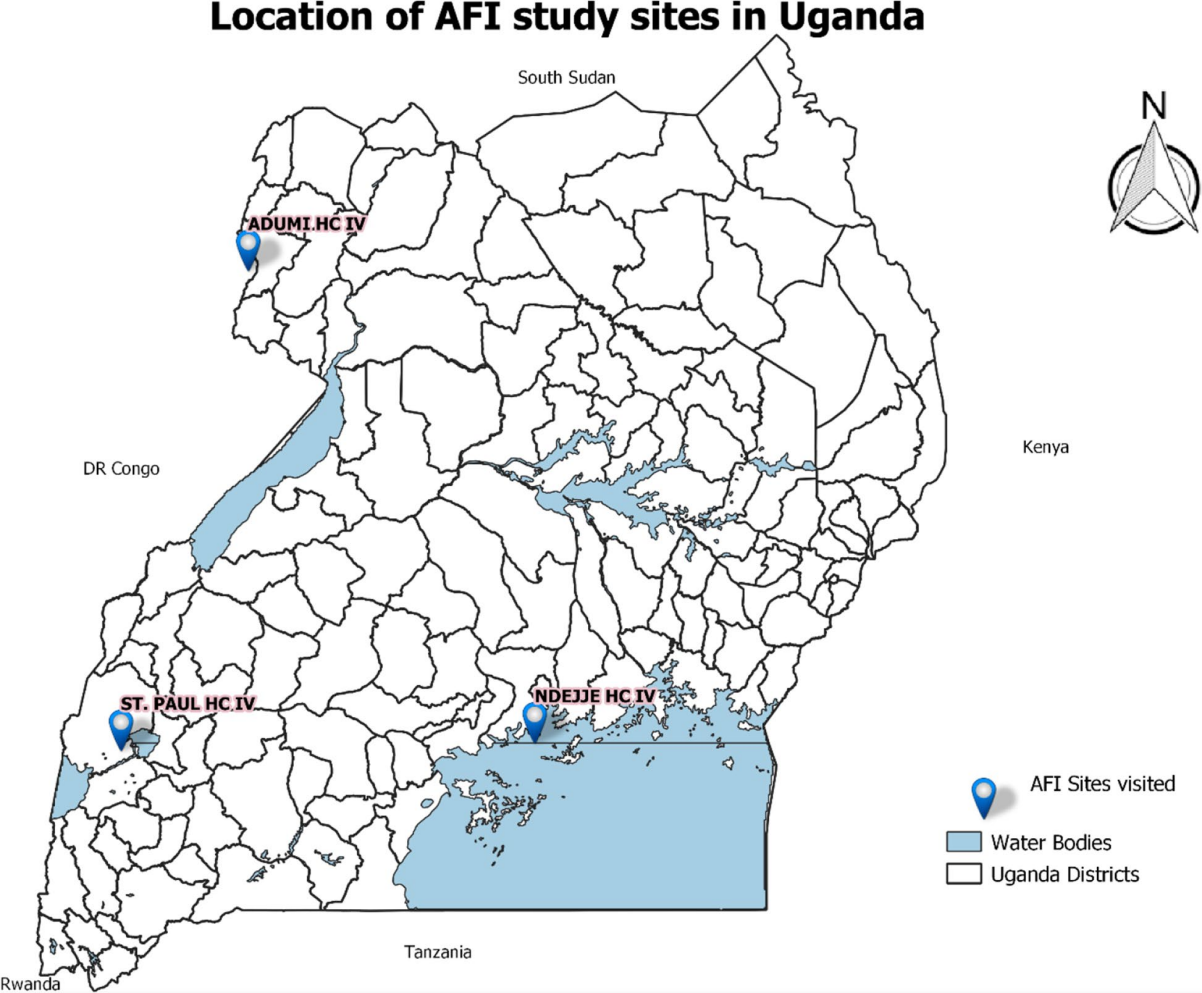
A map illustrating study clinics of the Uganda AFI prospective study

This study clinic is located in Kasese district, western region (Fig. 1), within the town of Kasese. The district is located along the Equator and borders Lake Edward to the south, Lake George to the east, and the Rwenzori Mountains and Democratic Republic of Congo (DRC) to the west. Kasese district had an estimated population of 694,992 in 2014 [22], with Kasese Municipality (pop. 101,557) being the main urban centre. The district is mainly agricultural, with over 85% of the population involved in subsistence farming. It has two distinct rainy seasons: March–May and October-December, with an average annual precipitation of around 930 mm. Temperatures are consistent throughout the year, with average daily lows and highs of 19 °C and 30 °C respectively [23].

#### Ndejje Health Centre IV (Coordinates: 00°14.150’ N 032°34.921’E; 3985 feet above sea level)

This study clinic is located in Makindye Ssabagabo Municipality (pop. 282,664), a highly urbanized area, within Wakiso district, in the central region (Fig. 1) [22]. Wakiso district has a population of over 2 million people and surrounds Kampala district, bordering Lake Victoria to the south and east. The district is urbanized with primary economic activities, including agriculture, fishing, manufacturing, sand mining, tourism, and commercial services. There are two distinct rainy seasons, the first from March to June and the second from August to November, with annual precipitation of about 1300 mm. There is a slight variation in temperature throughout the year, with an average daily low and high of 17 °C and 27 °C [24].

#### Adumi Health Centre IV (Coordinates: 03°02.316’N 30°49.994’E; 4052 feet above sea level)

This study clinic is located in Arua district, northern region (Fig. 1), in a rural area about 15 km west of Arua City. The district extends from the Albert Nile River valley in the east to the uplands and DRC to the west with a land area of 3234 km^2^. According to the 2014 Uganda National Population and Housing Census, the district had a population of 782,077, with Arua city (pop. 45,883) as the main urban center [22]. Arua district has experienced a high influx of refugees from South Sudan, which has stressed the natural environment and resources and has resulted in deforestation in the district [25]. Agriculture is the dominant economic activity, with cash crops including tobacco, nuts, fruit, and maize; animal husbandry includes goats, pigs, and fish. Local annual precipitation averages about 1400 mm; the rainy season runs from March to November, and peaks in May and October. Average daily low and high temperatures range from 17 °C and 27 °C respectively, from June to October, to 19 °C and 31 °C during January through March [26].

### Study participants’ recruitment and enrollment

Study participants were recruited from each study clinic’s outpatient department (OPD), based on a recruitment strategy. The number of patients recruited per district during the study was proportionately based on the total number of malaria cases diagnosed by the district in 2007 as reported in the Health Management Information System (HMIS) of UMoH. Potential participants were systematically screened for AFI from the OPD registers using a pre-determined interval (every nth patient) and invited to participate in the study after signing informed consent (Supplementary Table S1). If an individual declined participation, the next patient in the register was invited to be recruited in the study. This strategy ensured that recruitment was evenly distributed throughout the entire period of the study and accounted for periodic variations in-clinic patient load. It also ensured that the representation of the estimated undifferentiated AFI case population in the study region from which the subject was recruited was proportional to the whole study population. Based on sample size calculations, the study enrollment of a minimum of 1152 subjects allowed estimation of disease incidence ± 5% with 95% confidence.

All three study clinics concurrently recruited participants into the study. An oversampling of 15% was incorporated to account for the loss to follow-up. The inclusion criteria were as follows:

- Be at least two years of age with reported fever for at least two days and up to seven days, or temperature on admission of 38.5 °C or greater with no identified cause of fever, such as pneumonia or focal infection, or
- Be at least two years of age and suspected of having brucellosis or TF using the following definitions:
  Brucellosis: An illness characterized by acute or insidious onset, with continued, intermittent or irregular fever of variable duration, profuse sweating particularly at night, fatigue, anorexia, weight loss, headache, arthralgia, and generalized body aches.
  TF: An illness often characterized by insidious onset of sustained fever, malaise, headache, constipation or diarrhea, rose-colored spots on the chest, enlarged spleen and liver, anorexia, relative bradycardia, or nonproductive cough.

Participants were seen during the first (baseline) visit at the OPD, and a standardized questionnaire with patient’s demographic data, clinical presentation, and history (fever, headache, rash, abdominal pain, diarrhea, vomiting, bleeding from any site, anorexia, muscle and joint pains), and reported vector previous past exposure (such as ticks, lice, fleas, and mosquitoes) was completed by the study staff. To increase awareness, participants were informed of AFI etiologies and their risk factors during enrollment. Guidance for treatment was based on rapid diagnostic test (RDT) results provided to the participants’ healthcare providers (HCPs). A home or clinic follow-up visit was completed at least two weeks post-enrollment, during which an additional questionnaire assessed the patient’s follow-up clinical status and possible AFI risk factors, including housing characteristics, occupation, animal or livestock exposure, agricultural activities, use of mosquito nets, and water sources for consumption and other daily uses as well as handwashing practices.

To minimize loss to follow-up, study staff provided appointment cards prior to discharge at the baseline visit, made reminder telephone calls a few days prior to the follow-up visits, reimbursed travel expenses of returning participants, and when necessary, performed in-home follow-up for those who did not return to the clinic for follow-up. The clinic staff were responsible for all aspects of patient management and were managed according to the existing standard of care guidelines from the UMoH [27].

### Serum collection and laboratory methods

Study staff drew venous blood (approximately 5 ml) from each eligible enrollee at baseline and follow-up visits. Samples were centrifuged and sera aliquoted on the day of collection, and aliquots were stored at 2-8^0^C. Serum samples were screened at the time of patient’s visit for malaria, TF, brucellosis, and leptospirosis using their respective RDTs at study clinics. RDT results were provided immediately to clinic staff to inform patient management.

Aliquoted sera were transported at 2-8^0^C to the UVRI laboratory within one week to one month of collection for confirmatory testing as well as additional testing for diseases for which no RDT was utilized. Rickettsioses, chikungunya virus (CHIKV), dengue virus (DENV), and West Nile virus (WNV) testing were conducted at UVRI. Results of testing conducted at UVRI laboratory were provided to the clinic staff as they became available, supplementing RDT results to further guide patient diagnosis and treatment. For long-term storage at UVRI, sera were frozen at -80^0^C. Additional quality assurance testing for leptospirosis and rickettsiosis was conducted at CDC in Atlanta, GA, USA.

#### Malaria

Malaria testing in clinics was performed using Rapid 1–2-3 malaria HEMA Express Rapid Diagnostic Test (RDT) (Miramar, FL, USA), which tested for *Plasmodium falciparum*, *P. malariae*, and *P. vivax* on the baseline blood samples. Patients that were positive on the Rapid-1–2-3 malaria HEMA Express RDT were given diagnoses with malaria infection by the respective malaria parasite identified on the RDT cassette. A clinical laboratory evaluation of the Rapid 1–2-3 HEMA Pf (*Plasmodium falciparum*) assay conducted by UVRI reported a sensitivity of 75.0% and a specificity of 87.0%. For a subset of patients from Ndejje HC IV in the central region, thick and thin blood smears were examined to evaluate the performance of malaria RDT. Details of malaria RDT evaluation will be reported elsewhere.

#### Typhoid fever

Patients with a positive test on Tubex serology assay (IDL Biotech, Bromma, Sweden) were considered having TF (*S. enterica typhi*). The kit insert reported a sensitivity of 95% and sspecificity of 80%.

#### Brucellosis

Brucella IgM testing was performed at the baseline visit, while IgG testing was performed on convalescent serum at the follow-up visit, using the Brucella IgM and IgG Lateral Flow Assays (Life Assay Diagnostics, Cape Town, South Africa) respectively and following the manufacturer’s instructions. The assay was reported to have a combined IgM and IgG sensitivity of 96% and a specificity of 99% in a study done in Spain [28]. Confirmatory testing for brucellosis was done at UVRI using the CDC *Brucella* micro-agglutination test (BMAT), with minor modifications including the use of U-bottom plates, overnight incubation at 28 °C (for about 16 hrs), and discontinued use of safranin. Patients who tested positive on the Brucella IgM or Brucella IgG lateral flow assay and confirmed with BMAT were considered to have brucellosis.

#### Leptospirosis

Specimens were screened for leptospirosis per manufacturer’s instructions using the Leptospira IgM Lateral Flow Assay (Life Assay Diagnostics, Cape Town, South Africa). Patients with positive IgM lateral flow assay were considered to have acute infection. An evaluation study done in Andaman islands, off the coast of India, reported sensitivities of 52.9% during the first week of illness and 86% during weeks 2–4. The corresponding specificities were 93.6% and 89.4% [29]. All samples that tested positive for leptospirosis on the screening test and 20% of the negatives were sent to CDC in Atlanta, GA, USA for confirmation using the in-house Leptospira micro-agglutination test (LMAT). Briefly, live leptospiral cell suspensions representing 17 serogroups were incubated with serially diluted serum specimens. The resulting agglutination titers were read using darkfield microscopy. The reported titer was the highest dilution of serum that agglutinated at least 50% of the cells for each serovar tested [30].

#### Rickettsiosis

Rickettsia Immunofluorescent assays (Focus Diagnostics, Cypress, CA, USA) were used for detection and semi-quantitation of human IgG antibodies reactive with broad whole-cell antigens of representative members of the spotted fever group Rickettsia (SFGR, *R.rickettsii*) and typhus group Rickettsia (TGR, *R. typhi*). Because multiple spotted fever rickettsial species, as well as *R. prowazekii* (another typhus group species), can occur in Africa, we cannot be sure of the actual etiology for each patient utilizing the group-broad antigens. Reactivity in the assays can only be assigned to the group and not to that particular species. Confirmation of acute, recent, or past infection of spotted fever rickettsiosis and typhus fever was based on IgG endpoint titers at baseline and/or a comparison of titers from baseline and convalescent serum samples following the manufacturer’s instructions.

According to the kit manufacturer’s instructions, evidence of recent or current infection was confirmed when there was either fourfold or greater rise in IgG endpoint titer between the two paired samples (baseline and convalescent) tested in parallel or an IgG serum endpoint titer of 1:256 or greater with a single sample for the respective rickettsial sero group. Past infection was considered when a single IgG titer was between 1:64 and less than 256 or two paired samples whose titers do not give a fourfold increase in titer. Samples whose titration IgG end point titers were less than 1:64 were considered to be negative.

#### West Nile virus

Samples collected on the baseline visit were screened for WNV using West Nile Virus IgM Capture ELISA (FOCUS Diagnostics, Cypress, CA, USA), while the convalescent samples were tested using West Nile Virus IgG ELISA (FOCUS Diagnostics, Cypress, CA, USA) test. Samples that were West Nile virus IgM Capture ELISA positive are reported as acute WNV infection and those positive on West Nile Virus IgG ELISA as past infections. According to the manufacturer, the IgM kit had a sensitivity of 99% and specificity of 100%; and IgG kit had a sensitivity of 90.4% and specificity of 100%.

#### Chikungunya virus

Specimens were screened for CHIKV using the Chikungunya IgM ELISA kit (Standard Diagnostics, Kyonggido, Korea) to detect IgM antibodies to CHIKV. The manufacturer, reported this test as highly sensitive and specific (93.6% and 95.9%, respectively). Samples that tested positive on chikungunya IgM ELISA at baseline and/or follow-up visits are reported as acute CHIKV infection.

#### Dengue virus

Specimens collected from patients at the baseline visit were screened for DENV using the Dengue NS1 Ag ELISA (Standard Diagnostics, Kyonggido, Korea) detecting the dengue virus NS1 antigen. Patients that were positive on the dengue NS1 Ag ELISA were considered to have DENV infection. According to the manufacturer, the RDT kit had sensitivity of 93.3% and specificity of 98.9%.

#### Evaluation of Typhoid fever RDT

A subset of samples (*n*=200) from St. Paul’s HC IV in Kasese district where TF outbreaks had been previously reported [11] were to be evaluated by blood culture on McConkey and blood agar after inoculation into Oxoid blood culture bottles following incubation for 24–48 hrs.

### Climatic, environmental and GIS Data

The Uganda Ministry of Water & Environment Department of Meteorology and United States Oak Ridge National Laboratory provided environmental and climatic data. These included temperature (°C), rainfall (mm Hg), and humidity (%). Geographic Information System (GIS) coordinates (latitudes and longitudes) and ground elevation (feet) of the study participants’ residences were captured by hand-held Garmin GIS equipment. Details on data modelling investigating the relationship between climatic and environmental factors, and AFI disease occurrence and distribution will be presented elsewhere.

### Data management and analysis

Data were entered in Epi Info™ (version 3.5.2) [31] and analyzed using SAS version 9.3 (SAS Institute, Cary, NC) and STATA Version 13 (Stata Corp., College Station, TX, USA). Categorical variables were summarized using frequencies, percentages, and proportions for general descriptions. Bivariate analysis using Pearson’s chi-square test was used to evaluate associations between study participants’ characteristics and AFI etiologies and also detect if there were statistically significant differences between study participants with AFI etiologies who were lost to follow-up compared to those who were not lost to follow-up. Participants’ age, fever duration and body temperature were summarized using means, standard deviation (sd), median, and range. A two-sample t-test was used to establish if there were statistically significant differences in mean body temperature of study participants who reported fever resolution versus those who still had fever at the convalescent visit. Krukalis-Wallis test was used to detect if there were statistically significant differences in the median age of study participants who were lost to follow-up between the study clinics. Spearman’s correlation was used to test the correlation between indoor residual spraying (IRS), mosquito bed nets use, and number of malaria cases. A *p*-value of < 0.05 was considered statistically significant.

## Results

### Sociodemographic characteristics

**Fig. 2:**
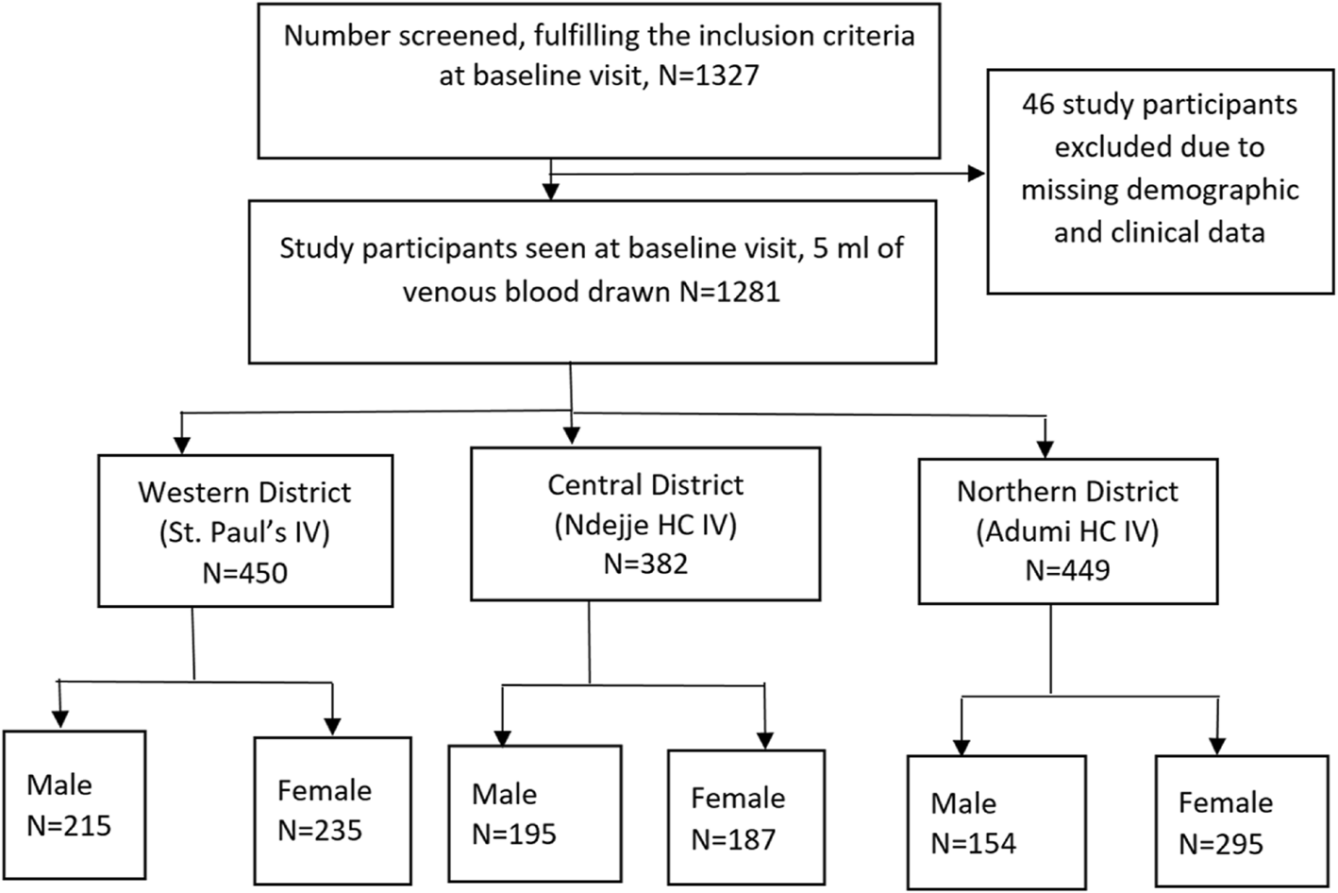
Study profile for participants recruited into the Uganda AFI study, April 2011 to January 2013

Overall, 1327 patients were screened at OPD during the baseline visit; 1281 (96.5%) of the participants were seen with complete sociodemographic and clinical information at baseline, and follow-up visits were included in the analysis. Of the 1281, 450 (35.1%), 382 (29.8%), and 449 (35.1%) were recruited from western, central, and northern regions, respectively (Fig. 2).

Fifty-six percent of the participants were female. The median age was 18 (range 2–93) years (Table 1), with age groups 1–10 and 11–20 years accounting for 55.9% of participants (Supplementary Table S2). Eighty (6.2%) of the participants seen at the baseline visit did not come back for the follow-up visit. There were statistically significant differences between study clinics in the median age and sex of participants who did not return at the convalescent visit (*p* = 0.001; *p* = 0.009, respectively), (Supplementary Table S3).

**Table 1:** Baseline sociodemographic characteristics of Uganda AFI study participants, April 2011 to January 2013

Variable	Total (%)	Western Region	Central Region	Northern Region
	***N* = 1281**	**(St. Paul’s HC IV)**	**(Ndejje HC IV)**	**(Adumi HC IV)**
		***N* = 450**	***N* = 382**	***N* = 449**
**Gender**				
**Male**	564 (44.0)	215 (38.1)	195 (34.6)	154 (27.3)
**Female**	717 (56.0)	235 (32.8)	187 (26.1)	295 (41.1)
**Age (years)**				
**Median (range)**	18 (2–93)	23.5 (2–60)	8 (2–60)	24 (2–93)
**Occupation**				
**Peasant farmer**				
**No**	945 (73.8)	376 (39.8)	379 (40.1)	190 (20.1)
**Yes**	336 (26.2)	74 (22.0)	3 (0.9)	259 (77.1)
**Student**				
**No**	765 (59.7)	285 (37.3)	118 (15.4)	362 (44.3)
**Yes**	516 (40.3)	165 (31.9)	264 (51.2)	87 (16.9)
**Health worker**^a^				
**No**	1249 (97.5)	421 (33.7)	382 (30.6)	446 (35.7)
**Yes**	32 (2.5)	29 (91.0)	0 (0)	3 (9.0)
**Merchant**^a^				
**No**	1160 (90.6)	419 (36.1)	363 (31.3)	378 (32.6)
**Yes**	121 (0.09)	31 (25.6)	19 (15.7)	71 (58.7)
**Office worker**^a^				
**No**	1241 (96.9)	416 (33.5)	378 (30.5)	447 (30.6)
**Yes**	40 (0.03)	34 (85)	4 (10.0)	2 (5.0)
**Unemployed**^a^				
**No**	974 (76.0)	387 (86.0)	336 (88.0)	251 (55.9)
**Yes**	307 (24.0)	63 (14.0)	46 (12.0)	198 (44.1)

^a^missing values

*N* = Total number

### Clinical characteristics

Of the 1281 participants who presented with undifferentiated fever at the baseline visit, 1054 (82.3%) had at least one AFI pathogen identified. Age-groups 2–10 and 11–20 years had over 50% of CHIKV, malaria, TF and SFGR infections (Supplementary Table S4). Malaria was diagnosed in 564 (44.0%) of the participants (Table 2). Of these, 404 (31.5%) had co-infections with other AFI pathogens, while 160 (12.5%) had malaria only (Table 3). Non-malarial pathogens were identified in 894 (69.8%) of the participants. These included rickettsioses, SFGR in 336 (26.2%) and TGR in 97 (7.6%); TF in 74 (5.8%); and arboviral infections, CHIKV in 716 (55.9%); WNV in 7 (0.5%); DENV in 10 (0.8%), (Table 2). Of note, only 2 (0.2%) participants were diagnosed with leptospirosis, and no brucellosis was identified. In 227 (17.7%) participants, no cause of infection was identified (Table 3). There were statistically significant differences between study participants with CHIKV who did not come back compared to those who came back during the convalescent visit (*p* = 0.008), (Supplementary Table S3). No statistically significant differences were observed with participants lost to follow-up who had tested positive for malaria, TF, SFGR and TGR on the baseline visit.

**Table 2:** Baseline clinical characteristics of Uganda AFI study participants, April 2011 to January 2013

**Laboratory diagnosis**	**Total (%)**	**Western Region**	**Central Region**	**Northern Region**	***p*-value**
	***N* = 1281**	**(St. Paul’s HC IV)**	**(Ndejje HC IV)**	**(Adumi HC IV)**	
		***N* = 450 (%)**	***N* = 382 (%)**	***N* = 449 (%)**	
**Malaria**^ca^					
Negative	714 (55.7)	271 (38.0)	206 (28.9)	237 (33.1)	0.095
Positive	564 (44.0)	179 (31.7)	174 (30.9)	211 (37.4)	
**Chikungunya virus**^a^					
Negative	562 (43.9)	216 (38.4)	159 (28.3)	187 (33.3)	0.139
Positive	716 (55.9)	232 (32.4)	222 (31.0)	262 (36.6)	
**Typhus Group Rickettsiosis (TGR)**^a^					
Negative	1051 (82.0)	332 (31.6)	308(29.3)	411 (39.1)	< 0.001
Past infection	90 (7.0)	32 (35.6)	32 (35.6)	26 (28.8)	
Current infection	97 (7.6)	63 (64.9)	31 (32.0)	3 (3.1)	
**Spotted Fever Group Rickettsiosis (SFGR)**^a^					
Negative	685 (53.5)	318 (46.4)	256 (37.4)	111(16.2)	< 0.001
Past infection	75(5.9)	16 (21.3)	16 (21.3)	43 (57.4)	
Current infection	336 (26.2)	50 (14.9)	63 (18.7)	223 (66.4)	
**Typhoid fever**^a^					
Negative	1204 (94.0)	410 (34.1)	364 (30.2)	430 (35.7)	0.001
Positive	74 (5.8)	40 (54.1)	15 (20.3)	19 (25.6)	
**Dengue virus**^a^					
Negative	1262 (98.6)	442 (35.0)	379 (30.1)	441 (34.9)	0.203
Positive	10 (0.8)	3 (30.0)	1 (10.0)	6 (60.0)	
**West Nile virus**^a^					
Negative	968 (75.6)	321 (33.2)	281 (29.0)	366 (37.8)	0.500
Past infection	58 (4.5)	25 (43.1)	20 (34.5)	13 (22.4)	
Acute Infection	7 (0.5)	3 (42.9)	3 (42.9)	1 (14.2)	
**Leptospirosis**^a^					
Negative	1267 (98.9)	449 (35.4)	377 (29.7)	441 (34.8)	0.382
Positive	2 (0.2)	1 (0.1)	1 (0.1)	0 (0)	
**Co-infections**^a^					
2 pathogens	426 (33.3)	130 (30.5)	112 (26.3)	184 (43.2)	ND
3 pathogens	131 (10.2)	36 (27.5)	31 (23.7)	64 (48.8)	ND
4 pathogens	19 (1.5)	7 (36.8)	9 (47.4)	3 (15.8)	ND
5 pathogens	1(0.1)	0 (0)	1 (100)	0 (0)	ND

*ND* Not done

N = Total number

^a^Missing values

^b^A total of 39 participants in the AFI study had SFGR and TGR dual infection with rickettsiosis. Of these 17 were from St. Paul’s HC IV; 19 Ndejje HC IV and 3 Adumi HC IV

^c^20 participants had *P.vivax* infection; (19 St. Paul’s HC IV; 1 Adumi HC IV)

**Table 3:** Laboratory diagnosis of Uganda AFI study participants, April 2011 to January 2013

AFI Diagnoses	Frequency	Percentage	Cumulative %
Malaria, CHIKV	224	17.5	17.5
CHIKV	217	16.9	34.4
Malaria	160	12.5	46.9
SFGR, CHIKV	100	7.8	54.7
Malaria, SFGR, CHIKV	71	5.5	60.2
SFGR	58	4.5	64.7
Malaria, SFGR	50	3.9	68.6
TGR	20	1.5	70.1
TF	19	1.5	71.6
SFGR, TGR, CHIKV	15	1.2	72.8
TF, CHIKV	14	1.0	73.8
Malaria, TGR, CHIKV	13	1.0	74.8
TGR, CHIKV	11	0.8	75.6
Malaria, SFGR, TGR, CHIKV	10	0.8	76.4
Malaria, TF, CHIKV	9	0.7	77.1
SFGR, TF, CHIKV	8	0.6	77.7
Malaria, TF	6	0.4	78.1
Malaria, SFGR, TGR	5	0.4	78.5
TGR, TF	5	0.4	78.9
SFGR, TGR	5	0.4	79.3
Malaria, TGR	4	0.3	79.6
Malaria, SFGR, TF, CHIKV	3	0.2	79.8
SFGR, TF	3	0.2	80
DENV, CHIKV	3	0.2	80.2
Malaria, DENV, CHIKV	2	0.2	80.4
SFGR, DENV, CHIKV	2	0.2	80.6
TGR, DENV, CHIKV	2	0.2	80.8
WNV, CHIKV	2	0.2	81
TGR, TF, CHIKV	1	0.1	81.1
TF, WNV, CHIKV	1	0.1	81.2
TF, Leptospirosis, CHIKV	1	0.1	81.3
SFGR, TGR, TF, WNV	1	0.1	81.4
SFGR, TGR, TF, CHIKV	1	0.1	81.5
Malaria, WNV, CHIKV	1	0.1	81.6
Malaria, TGR, TF, CHIKV	1	0.1	81.7
Malaria, TGR, TF	1	0.1	81.8
Malaria, SFGR, WNV, CHIKV	1	0.1	81.9
Malaria, SFGR, TGR, WNV, CHIKV	1	0.1	82
Malaria, SFGR, TGR, CHIKV	1	0.1	82.1
Malaria, SFGR, DENV, CHIKV	1	0.1	82.2
Leptospirosis	1	0.1	82.3
No pathogen identified	227	17.7	100
Total	1281	100	

*TF* Typhoid fever, *WNV* West Nile virus, *CHIKV* Chikungunya virus, *DENV* Dengue virus, *SFGR* Spotted Fever Group Rickettsia, *TGR* Typhus Group Rickettsia

**Fig. 3:**
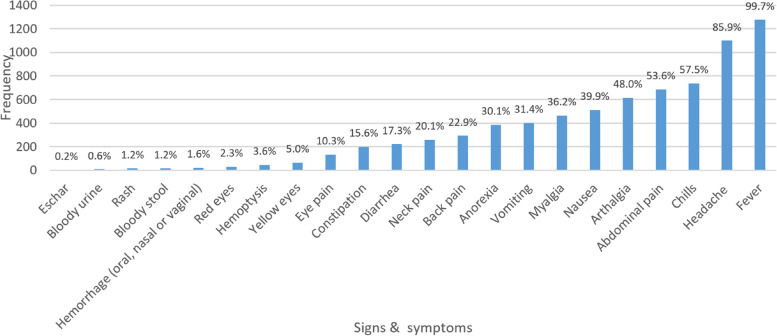
Clinical presentation during baseline visit of AFI study participants, April 2011 to January 2013

Participants presenting with a fever had a mean temperature of 37.9 °C (sd = 1.05 °C) and a fever duration of 3.8 days (sd = 2.2 days). Other predominant symptoms included headache, chills, abdominal pain, arthralgia, nausea, and myalgia (Fig. 3).

### Concurrent infections

Concurrent infections were common (Tables 2 and 3); overall, 577 (45.0%) participants had co-infection with more than one AFI pathogen. In 404 (31.5%) participants, evidence of co-infection with malaria and one or more non-malarial AFI pathogens was noted; 10 (0.8%) showed evidence of another arboviral co-infection with DENV. One hundred seventy-five (13.7%) participants had non-malarial AFI pathogen co-infection evidence, and of note, 39 (3.0%) participants presented with apparent SFGR and TGR co-infection.

### Occurrence and distribution of AFI pathogens

**Fig. 4:**
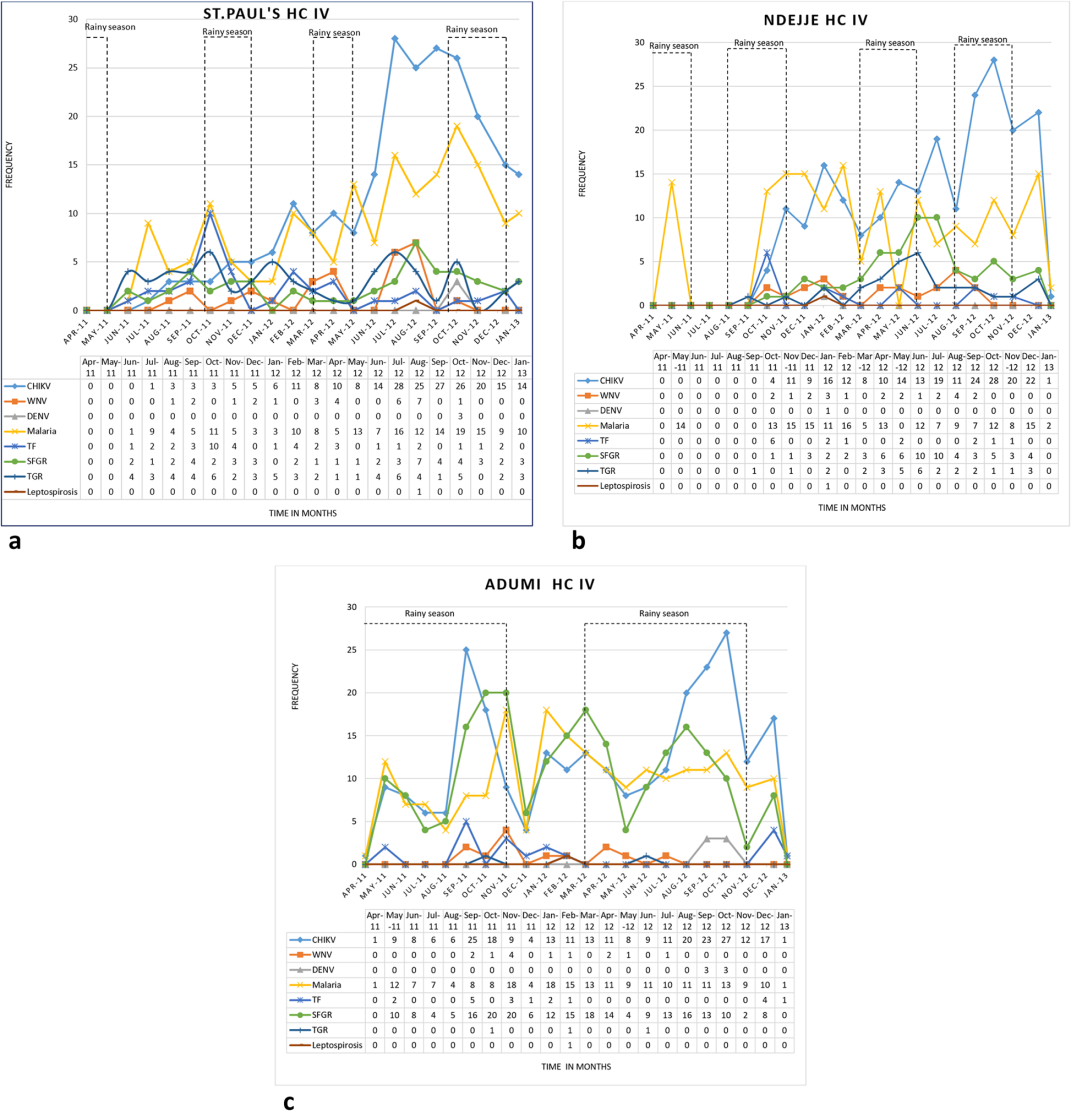
**a** Seasonal occurrence of AFI pathogens at St. Paul’s HC IV, April 2011 to January 2013. CHIKV: Chikungunya virus. HC: Health Centre. TF: Typhoid fever. SFGR: Spotted Fever Group Rickettsia. DENV: Dengue virus. TGR: Typhus Group Rickettsia. WNV: West Nile virus. **b** Seasonal occurrence of AFI pathogens at Ndejje HC IV, April 2011 to January 2013. CHIKV: Chikungunya virus. HC: Health Centre. TF: Typhoid fever. SFGR: Spotted Fever Group Rickettsia. DENV: Dengue virus. TGR: Typhus Group Rickettsia. WNV: West Nile virus. **c** Seasonal occurrence of AFI pathogens at Adumi HC IV, April 2011 to January 2013. CHIKV: Chikungunya virus. HC: Health Centre. TF: Typhoid fever. SFGR: Spotted Fever Group Rickettsia. DENV: Dengue virus. TGR: Typhus Group Rickettsia. WNV: West Nile virus

There were statistically significant differences in the occurrence and distribution of some of the investigated AFI pathogens at the study clinics (Table 2). TF and TGR were more frequently observed in the western region, while SFGR were more frequently diagnosed in the northern region (Table 2). Rainy season was associated with highest number of TF participants recruited while TGR and SGFR highest peaks were observed towards the end of rainy season and during dry season. No seasonality was observed with malaria, CHIKV, WNV, DENV and leptospirosis. Malaria and CHIKV disease occurrence had similar trends (Fig. 4a-c).

Of the 1281 participants, 1182 (92.3%) reported having seen an ectoparasite in the last month; 705 (55.0%) had slept under mosquito nets the night prior to consultation at the study clinics, and 25 (2.0%) had the interior walls of their dwellings sprayed against mosquitoes in the last 12 months prior to this study (Table 4).

**Table 4:** Baseline clinical characteristics of prior exposure to ectoparasites, use of mosquito nets and indoor residual spraying in dwellings of Uganda AFI study participants, April 2011 to January 2013

Exposure	Total (%) *N* = 1281	Western Region (St. Paul’s HC IV) *N* = 450	Central Region (Ndejje HC IV) *N* = 382	Northern Region (Adumi HC IV) *N* = 449	*p*-value
**Lice**					
**No**	1226 (95.7)	409 (33.4)	382 (31.1)	435 (35.5)	*p* < 0.001
**Yes**	55 (4.3)	41 (74.5)	0	14 (25.5)	
**Fleas**					
**No**	1133 (88.4)	429 (37.9)	375 (33.1)	329 (29.0)	*p* < 0.001
**Yes**	148 (11.6)	21 (14.2)	7 (4.7)	120 (81.1)	
**Ticks**					
**No**	1061 (82.8)	350 (33.0)	379 (35.7)	332 (31.3)	*p* < 0.001
**Yes**	220 (17.2)	100 (45.5)	3 (1.4)	117 (53.1)	
**Mosquitoes**					
**No**	108 (8.4)	5 (4.6)	98 (90.8)	5 (4.6)	*p* < 0.001
**Yes**	1173 (91.6)	445 (37.9)	284 (24.2)	444 (37.9)	
**Ectoparasite seen in the last one month**^a^					
**No**	98 (7.7)	3 (3.1)	93 (94.9)	2 (2.0)	*p* < 0.001
**Yes**	1182 (92.3)	447 (37.8)	288 (24.4)	447 (37.8)	
**Slept in a mosquito net last night**^a^					
**No**	412 (32.2)	118 (28.6)	146 (35.4)	148 (36.0)	*p* < 0.001
**Yes**	705 (55.0)	284 (40.3)	197 (27.9)	224 (31.8)	
**Interior walls of the dwelling sprayed against mosquitoes**^a^					
**No**	1104 (86.2)	392 (35.5)	327 (29.6)	385 (34.9)	*p* = 0.001
**Yes**	25 (2)	10 (40.0)	15 (60.0)	0 (0)	

^a^missing values N = Total number

Increased use of IRS was weakly correlated with decreased malaria cases (rho = -0.10, *p* = 0.04); mosquito net use was not correlated with a decrease in malaria cases (rho = -0.01, *p* = 0.10)

### Evaluation of Typhoid fever RDT

Only 51 (25.5%) samples were collected for blood culture, and all tested negative for TF.

Because there was potential for the internal validity of the study to be compromised due to delays in sample collection from the study clinic to the hospital laboratory for blood culture; these results were therefore discarded.

### Treatment and clinical outcomes

Information on clinical outcomes is available from 1125 (87.8%) of the participants. Of 1125 participants, 1087 (96.6%) reported both improvement on treatment and fever resolution, during the convalescent visit (Table 5). The mean temperature of participants who reported no fever resolution was higher than those who reported fever resolution at the convalescent visit, respectively; 37.0 ^0^C (sd = 0.6) Vs 36.5 ^0^C (sd = 0.9), *p*-value = 0.003.

**Table 5:** Clinical outcomes of Uganda AFI study participants, April 2011 to January 2013

		Reported fever resolution	Reported no fever resolution	Total
Improvement on treatment	Reported improvement	1087	32	1119
	Reported no improvement	4	2	6
	Total	1091	34	1125

## Discussion

This collaborative study reveals that numerous pathogens contribute to the AFI burden in all three study regions. Overall, one or more AFI potential pathogens were diagnosed in more than 8 out of 10 study participants presenting with undifferentiated fever during the study period. The proportion of participants with acute arboviral infections and rickettsioses (TGR, SFGR) was relatively higher than in studies done in Tanzania [6], Senegal [32], and Nigeria [33]. These findings may be attributable to Uganda’s unique biologic diversity and rising population density that may, in turn, favor higher rates of vector-borne transmission of pathogens from animals to humans [34, 35].

To the best of our knowledge, this is the first study in SSA to investigate the etiology of AFI among outpatient and ambulatory patients recruited from all age-groups with excellent clinical outcomes, on such a broad range of pathogens in several distinct regions differing by climate, environment, and population density [22, 24, 26]. Most of the studies done elsewhere have investigated the etiology of AFI among hospitalized patients [1, 6, 36, 37] and some reporting poor prognosis [1]. In the current study, we demonstrate that arboviral, rickettsial, and typhoid infections, in addition to malaria, contribute to > 80% of the AFI disease burden in the study regions. However, research published after the conclusion of this study demonstrated long-term persistence of IgM antibodies in flavivirus infections and high levels of IgM and IgG cross-reactivity between flaviviruses [38, 39]. Consequently, samples that were West Nile virus IgM Capture ELISA positive in this study have since been determined to be inconclusive for acute WNV infection.

CHIKV RDTs validation in studies done elsewhere demonstrated much lower sensitivity and specificity (30% and 73%, respectively), likely due to cross-reactivity with other alphaviruses [40]. Evidence of cross-reactivity between CHIKV and O’nyong-nyongo virus (ONNV), another alphavirus that was first identified from northern Uganda in 1959 [41] has been documented [42]. CHIKV and ONNV infections present with similar clinical characteristics that make definitive disease diagnosis difficult without appropriate diagnostic assays [43]. In one serosurvey of healthy blood donors, CHIKV had the highest antibody prevalence, but plaque reduction neutralization test (PRNT) results suggested ONNV as the infecting virus for a majority of the samples [42]. Thus, serological confirmation of CHIKV or ONNV infection requires a PRNT to determine the ability of an antibody sample to neutralize each virus [42]. Additionally, the hallmark of CHIKV clinical presentation is fever, prolonged-debilitating arthralgia and a body rash [43]. In our study, 43.9% of the samples from participants tested positive on chikungunya IgM ELISA test without PRNT confirmation. Taken together with evidence of previous reemergence in Uganda [44], it is likely ONNV was the AFI causative pathogen since a large proportion of participants with fever presented with non-prolonged and non-debilitating arthralgia (48.0%) and only 1.2% of these participants had a body rash. Furthermore, IgM persists for alphaviruses up to 10 months [45] and thus, samples that tested IgM positive may not reflect acute infection.

This study revealed that 69.7% of the participants had evidence of non-malarial AFI pathogen infection. Rickettsial infections and arboviral infections were the predominant etiologies. Limited data on CHIKV [46, 47], WNV [48], and TGR [17] exist in Uganda, and most studies were conducted 40 years or more prior to this study. This is the first report of SFGR among Ugandan patients presenting at health facilities with acute undifferentiated fever. Previously, SFGR has been isolated in ticks in Uganda but not in AFI patients [16, 49]. Our findings confirm that arthropod vector-borne infections are diseases of major clinical and public health importance in Uganda. Thus, clinicians must adopt a new paradigm and shift from presumptive management of undifferentiated AFI as malaria to performing appropriate diagnostic testing in health settings and using the results as treatment basis [50].

In our study, malaria was confirmed by laboratory testing in 44% of the participants. By contrast, a similar study among hospitalized AFI patients in northern Tanzania reported that malaria was diagnosed clinically in 60.7% but confirmed in only 1.6% [6]. We observed that only 55.0% of the participants were using bednets, and 2.0% of residences had received indoor residual spraying (IRS) during 12 months prior to the study, suggesting a very low coverage; this likely contributed to the high rate of malaria infections observed. In order for IRS to be effective, high coverage of ≥ 80% has been recommended by the World Health Organization (WHO) and has been associated with a 65% reduction in malaria infections [51]. WHO recommends long-lasting insecticidal nets (bednets) and IRS as the most effective vector control interventions; bednets protect individuals against infective malaria bites while IRS involves coating the walls and other surfaces of a house with a residual insecticide which kills mosquitoes that come in contact with these surfaces [52]. A significant reduction in malaria morbidity has been documented in northern and eastern Uganda, where IRS and bednet interventions have been implemented [53, 55].

Our study found that concurrent infections were common, with up to 45.0% of the participants having evidence of infection with more than one AFI-associated pathogen. Similar findings have been observed elsewhere in Africa for malarial [32, 33], and non-malarial [56] co-infections. In our study, 26.4% of the participants had malaria with arboviral co-infections, while non-malarial co-infections were seen in 13.7% of participants. It is possible that CHIKV and WNV are commonly circulating arboviruses that have been unrecognized due to the lack of diagnostic capacity; thus, more definitive testing such as confirmation by PRNT or Real-Time Polymerase Chain Reaction (RT-PCR) is necessary to confirm this. A study conducted in Senegal found evidence of CHIKV in a pool of field-collected *Anopheles funestus* [57], and *A. funestus* is also one of the principal vectors for *P. falciparum* in Uganda [58, 59]. Therefore, it is theoretically plausible that participants may have been bitten by *A. funestus* mosquitoes harboring both malaria parasites and an arbovirus, resulting in dual infection. Alternatively, consecutive bites from two infected mosquitoes or species (e.g. *Anopheles* spp. for malaria and *Aedes* spp. for arboviruses) as postulated by Sow et al. could also have resulted in the dual infection [32].

There were statistically significant variations in the occurrence and distribution of some AFI diseases studied between study clinics. TF and TGR were identified more frequently in Kasese district (western region). In contrast, SFGR was more frequent in the northern region in Arua district. Variations in climate and environment in the areas where these clinics are located may account for this finding. The southwestern region, especially Kasese district, is characterized by frequent TF outbreaks [11, 60]. In our study we observed that rainy season was associated with the highest number of TF cases. Similar observations have been made with other studies done in Uganda [60, 61]. Precipitation and flooding, fecal environmental contamination, and limited water treatment have been identified as risk factors [60]. We also found out that the highest monthly number of TGR and SFGR patients recruited in the study occurred during the end of the rainy and dry seasons. Participants with TGR were predominantly identified in the central and western regions; both clinics serve patients living in predominantly urbanized settings, which may serve as a suitable habitat for rats. Rats are hosts to the rat flea (*Xenopsylla cheopsis*), a common vector of murine typhus [62]. Risk factors of warmer temperatures and lower population density for SFGR have been reported elsewhere [63]; the more rural population and seasonally higher average temperatures associated with the northern district may present a more favorable environment for SFGR than other clinics locations. Climatic factors data modelling and environmental factors spatial modelling could provide further insight of AFI disease incidence and help predict other areas of higher incidence in Uganda.

Brucellosis has been recognized as a significant cause of AFI in some regions of Uganda; for example, a prevalence of 14.9% was reported among pastoralists in a rural community hospital in Southwestern Uganda [64]. However, brucellosis was not observed in participants from any of the three study clinics, likely due to low rates of cattle husbandry and lower consumption of raw milk in the study areas compared with high prevalence districts in Uganda [64, 67]. Similarly, only two cases of leptospirosis were identified, one each in the central and western regions. In contrast, a seroprevalence of 35% for leptospirosis was reported among individuals presenting to two clinics in Hoima district, also in western Uganda midway between Arua and Kasese districts [68]. Based on these conflicting results, further studies examining the burden of brucellosis and leptospirosis in Uganda are indicated.

### Study limitations

This study had several limitations. (1) Reporting of the ectoparasites' past exposure status during interviews by enrolled participants was a potential source of recall bias. This was minimized by providing charts with photographs of the ectoparasites and limiting the period of exposure not exceeding one month prior to their clinic visit. (2) Using a stringent case definition with inclusion criteria for body temperature of ≥ 38.50 C but not ≥ 38.00 C, a commonly used threshold, the current study was amenable to selection bias. In addition, the recruitment of only outpatient and ambulatory patients was also another potential source of selection bias as excluded ill and hospitalized patients with AFI etiologies. It could be that the spectrum of infections among inpatient patients was different from what we observed in our study. (3) The possible serologic cross-reactivity within the arboviruses families might have led to non-differential misclassification bias of outcomes (with bias of risk ratios towards the null) for some of the arboviruses (i.e. CHIKV) leading to overestimation of the true burden of arboviruses [69]. We were unable to distinguish between patients with possible, for example, CHIKV and ONNV [42], WNV and DENV [70] due to previously reported serologic cross-reactivity within arbovirus families as confirmatory testing with PRNT or RT-PCR was not performed because of resource-limitations. PRNT is able to measure virus-specific neutralizing antibodies and determine the cause of the primary infection with high specificity [71] and clarify cross-reacting results; however, since the test is expensive and very labor-intensive, was not available for use in this study. RT-PCR is effective in diagnosing arboviruses by differentiating between families with low occurrence of cross-reactivity and low nonspecific reactions. Additionally, the technique is able to identify the pathogens during the viremia stage during the early stage of the disease [72]. (4) A limited number of pathogens were investigated due to limited resources, including laboratory diagnostic capacity. For example, a laboratory-confirmed outbreak of Rift Valley fever (RVF), which can present as AFI, was confirmed in the southern region of Uganda three years after the end of this study [73]; however, RFV investigations were not included in this study due to resource limitations. Additionally, we did not investigate bacterial and fungal bloodstream infections, which are HIV-infection related, although have been reported as significant causes of sepsis, contributing to the AFI disease burden [1, 36]. In our study, we targeted HIV-negative patients and may have included those with unknown HIV results since we did not test for HIV or did not report on the HIV status of febrile patients. A recent study done in Uganda has identified tuberculosis as a predominant cause of AFI among HIV-infected and hospitalized patients [36]. (5) The cause of infection in 17.7% remains unknown, even after testing for multiple pathogens due to diagnostic capacity limitations leading to underestimation of the AFI disease burden. Metagenomic next-generation sequencing is a new diagnostic technology and has been recently used to identify pathogens in patient samples that are negative by all other diagnostics, to better understand the causes of AFI [74, 75]. This assay may not be routinely in use for the majority of clinical settings in SSA due to cost implications. (6) Of the 1281 participants with undifferentiated AFI, 6.2% did not participate in a follow-up visit. This loss to follow-up could have resulted from recovery from illness after receiving treatment following the baseline visit and, therefore, participants feeling no need to continue participating in the study. However, it is anticipated that the loss to follow-up may have affected the generalizability of the study findings in CHIKV participants who were lost to follow-up. In addition, where paired baseline and convalescent serum samples were required to confirm AFI infection, this was not possible and could have led to underestimation of AFI burden. (7) The current study was completed almost a decade ago, and there is a possibility of an epidemiology shift of AFI pathogens reported due to the changes in climate, environment and population density, including the introduction of vector control activities in the study regions. However, recent studies confirm that malaria [76] and arboviral [77, 78], and typhoid infections [60] are still significant contributors to the AFI disease burden in Uganda. No recent information on rickettsiosis, chikungunya and West Nile Fever has been published among acute febrile patients in health facilities. (8) There were delays in specimen testing and transportation due to the extra workload on clinic staff who had routinely assigned duties as well as study-related tasks. Collected sera were often kept at 2-8^0^C in refrigerators at the study clinics for several days before shipment to UVRI. The refrigerators were subject to occasional power outages without generator backup. Fluctuation in storage temperatures might have led to potential specimen degradation and affected the internal validity of the study findings.

## Conclusion

This collaborative study shows that malaria and rickettsioses are major causes of AFI in selected clinics in the central, northern, and western regions of Uganda. Arboviral infections and TF in some regions may contribute a substantial burden to AFI as well. Further research and development of non-malarial AFI serological tests could improve the etiologic diagnosis of AFI, though, implementation of serological tests may be costly and complex in a limited-resource setting. The development of a cost-effective multiplex point-of-care testing is a future prospect and has been proposed by Yager P et al*.* [79]. This could reduce testing turn-around time by quickly identifying the etiology from a pathogen group causing similar symptoms and would have substantial public health relevance. Based on findings from this study, infectious diseases that contribute to AFI may be amenable to multiplexing include malaria, TF, SFGR, and TGR; inclusion of testing for specific arboviral infections may prove to be more problematic. In the meantime, there is a need for continued investigation of AFI in other regions of Uganda, based on differences in climate, environment, population density, and land use. Entomological studies evaluating vector competence and interventions aimed at vector control could augment these investigations and help better understand and mitigate the burden of AFI in Uganda.

## Supplementary Information

**Additional file 1: Table S1.** Recruitment strategy of AFI study participants based on malaria cases in 2007.

**Additional file 2: Table S2.** Age-group distribution of AFI study participants, April 2011 to January 2013.

**Additional file 3: Table S3.** Effect of loss to follow-up on study validity.

**Additional file 4: Table S4.** Diagnoses by age-group distribution, April 2011 to January 2013.

## Data Availability

Data may be accessed through the following link below; https://www.uvri.go.ug/sites/default/files/files/AFI%20data%20set%20%28web%29_shared.xls
