# Supplementary material for: Investigating the etiology of acute febrile illness: a prospective clinic-based study in Uganda
Source: BMC Infect Dis. 2023 Jun 16;23:411. doi: 10.1186/s12879-023-08335-4 (PMC10276394; doi:10.1186/s12879-023-08335-4)
Supplement: Supplementary file 1 — Additional file 1: Table S1. Recruitment strategy of AFI study participants based on malaria cases in 2007. [file 12879_2023_8335_MOESM1_ESM.pdf]

Table S1. Recruitment strategy for AFI participants based on malaria cases in 2007

**District**	**Study Clinic**	**Malaria clinical diagnoses per district, 2007**	**Per District % of Total Malaria Diagnoses**	**# Subjects per Study District (proportional to total population)**	**Oversampling by 15%**	**Rate for Subject Selection per District set at every *n*th eligible subject, where *n* =**
Arua	Adumi HC IV	272,591	272,591/ 926,445	0.294	(294 x 115)/100 =339	(Adumi HC IV total malaria case count for 2007)/339 (15,127 cases)/339: Every 44th patient (amended to every 21st patient). To a maximum of 29 (313/12) patients/month.
Wakiso	Ndejje HC IV	286,131	286,131/ 926,445	0.309	(309 x115)/100 =356	(Ndejje HC IV total malaria case count for 2007) / (11037 cases)/356: Every 31st patient. To a maximum of 30 (356/12) patients/month.
Kasese	St. Paul's HC IV	367,723	367,723/ 926,445	0.397	(397x115)/100 =457	(St. Paul's HC IV total malaria case count for 2007) /457 (4622 cases)/457: Every 10th patient. To a maximum of 38 (457/12) patients/month.
TOTAL		926,445	100	1.000	1152	

HC = Health Centre
