# Supplementary figures and images for: Investigating the etiology of acute febrile illness: a prospective clinic-based study in Uganda
Source: BMC Infect Dis. 2023 Jun 16;23:411. doi: 10.1186/s12879-023-08335-4 (PMC10276394; doi:10.1186/s12879-023-08335-4)

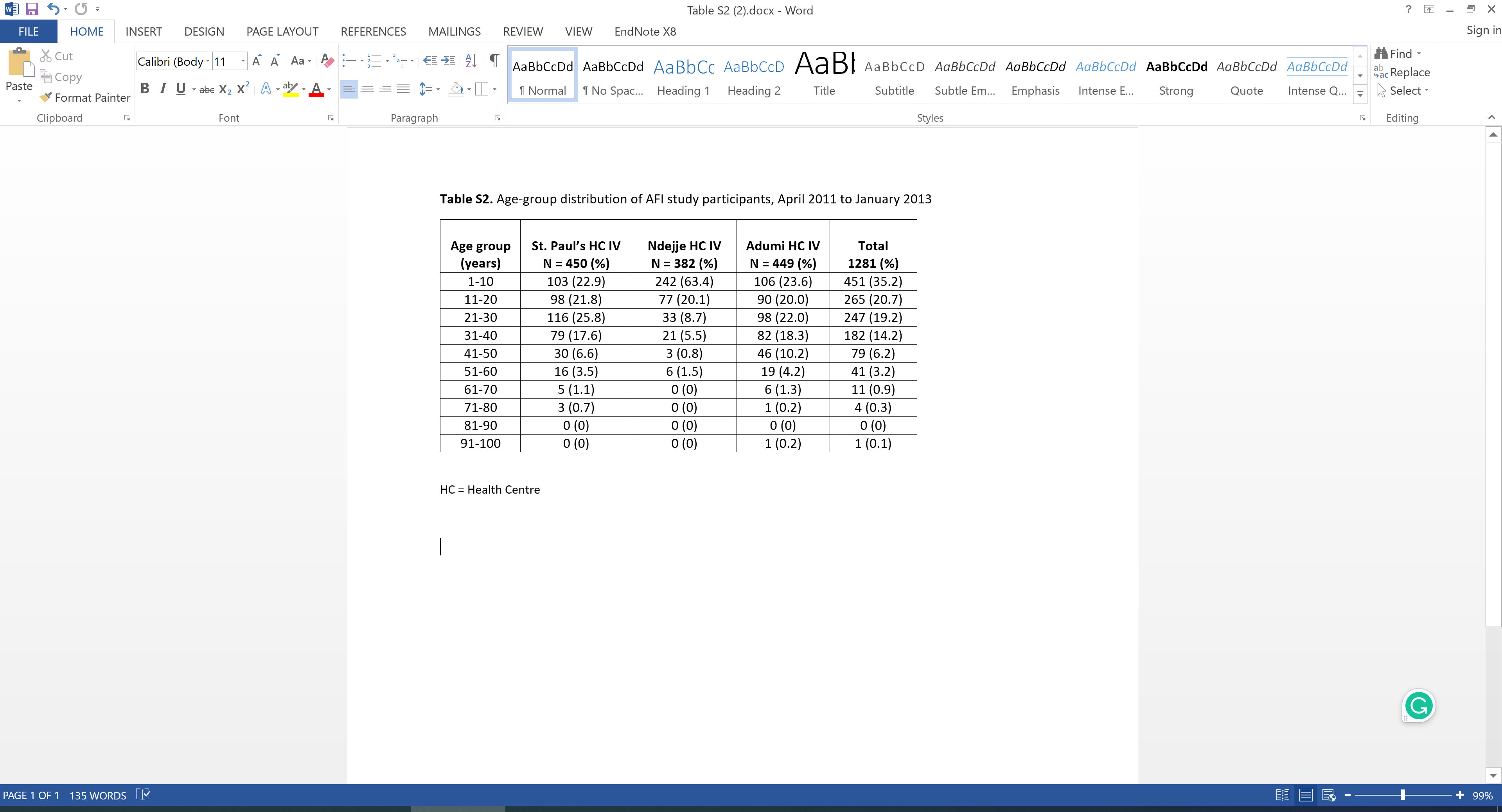

Supplement: Supplementary file 2 — Additional file 2: Table S2. Age-group distribution of AFI study participants, April 2011 to January 2013. [file 12879_2023_8335_MOESM2_ESM.docx]
