# Supplementary material for: Investigating the etiology of acute febrile illness: a prospective clinic-based study in Uganda
Source: BMC Infect Dis. 2023 Jun 16;23:411. doi: 10.1186/s12879-023-08335-4 (PMC10276394; doi:10.1186/s12879-023-08335-4)
Supplement: Supplementary file 3 — Additional file 3: Table S3. Effect of loss to follow-up on study validity. [file 12879_2023_8335_MOESM3_ESM.pdf]

Table S3. Effect of loss to follow-up of AFI participants on the study validity

Variable	St. Paul's HC IV	Ndejje HC IV	Adumi HC IV	p-value
Age (years)				
Median	23.5	6	28	0.0001[a]
Mean	21.1	8.2	26	
Observations (Rank sum)	15 (601.0)	25 (564.0)	40 (1916.0)	
Sex				
LTF Male	6	13	7	0.009[b]
LTF Female	8	12	34	
Malaria				
Positive No LTF	173	161	199	0.234[b]
Positive LTF	6	13	12	
TGR				
Positive No LTF	60	31	3	0.434[b]
Positive LTF	3	0	0	
SGFR				
Positive No LTF	49	61	205	0.148[b]
Positive LFT	1	2	18	
TF				
Positive No LTF	39	14	19	0.489[b]
Positive LTF	1	1	0	
CHIKV				
Positive No LTF	228	217	245	0.008[b]
Positive LTF	4	5	17	

HC = Health Centre

TGR = Typhus Group Rickettsia

SFGR = Spotted Fever Group Rickettsia

TF =Typhoid Fever

CHIKV = Chikungunya virus

No LTF = Not lost to follow-up

LTF = Lost to follow-up

[a]Kruskal-Wallis test

[b]Pearson chi-square test
