# Supplementary material for: Investigating the etiology of acute febrile illness: a prospective clinic-based study in Uganda
Source: BMC Infect Dis. 2023 Jun 16;23:411. doi: 10.1186/s12879-023-08335-4 (PMC10276394; doi:10.1186/s12879-023-08335-4)
Supplement: Supplementary file 4 — Additional file 4: Table S4. Diagnoses by age-group distribution, April 2011 to January 2013. [file 12879_2023_8335_MOESM4_ESM.pdf]

Table S4. Diagnoses by age-group distribution for AFI study participants, April 2011 to January 2013

Age-group (years)	CHIKV N = 716 (%)	WNV N = 63 (%)	DENV N = 10 (%)	Malaria N = 564 (%)	TF N = 74 (%)	SGFR N = 336 (%)	TGR N = 97 (%)	Leptospirosis N = 2 (%)
2-10	228 (31.8)	13 (20.6)	1 (10.0)	245 (43.4)	41 (55.4)	73 (21.7)	21 (21.6)	2 (100)
11-20	178 (24.9)	12 (19.0)	3 (30.0)	131 (23.2)	18 (24.3)	56 (16.7)	28 (28.9)	0 (0)
21-30	131 (18.3)	15 (23.8)	2 (20.0)	93 (16.5)	7 (9.5)	76 (22.6)	24 (24.7)	0 (0)
31-40	111 (15.5)	13 (20.6)	1 (10.0)	57 (10.1)	4 (5.4)	61 (18.2)	6 (6.2)	0 (0)
41-50	40 (5.6)	3 (4.8)	2 (20.0)	20 (3.5)	1 (1.4)	38 (11.3)	10 (10.3)	0 (0)
51-	28 (3.9)	7 (11.1)	1 (10.0)	18 (3.2)	3 (4.0)	32 (9.5)	8 (8.2)	0 (0)

CHIKV = Chikungunya virus

WNV = West Nile virus

DENV = Dengue Virus

TF = Typhoid fever

SGFR = Spotted Group Fever Rickettsia

TGR = Typhus Group Rickettsia
